# Supplementary material for: Decoding the spatial chromatin organization and dynamic epigenetic landscapes of macrophage cells during differentiation and immune activation
Source: Nat Commun. 2022 Oct 4;13:5857. doi: 10.1038/s41467-022-33558-5 (PMC9532393; doi:10.1038/s41467-022-33558-5)
Supplement: Supplementary file 1 — Supplementary Information [file 41467_2022_33558_MOESM1_ESM.pdf]

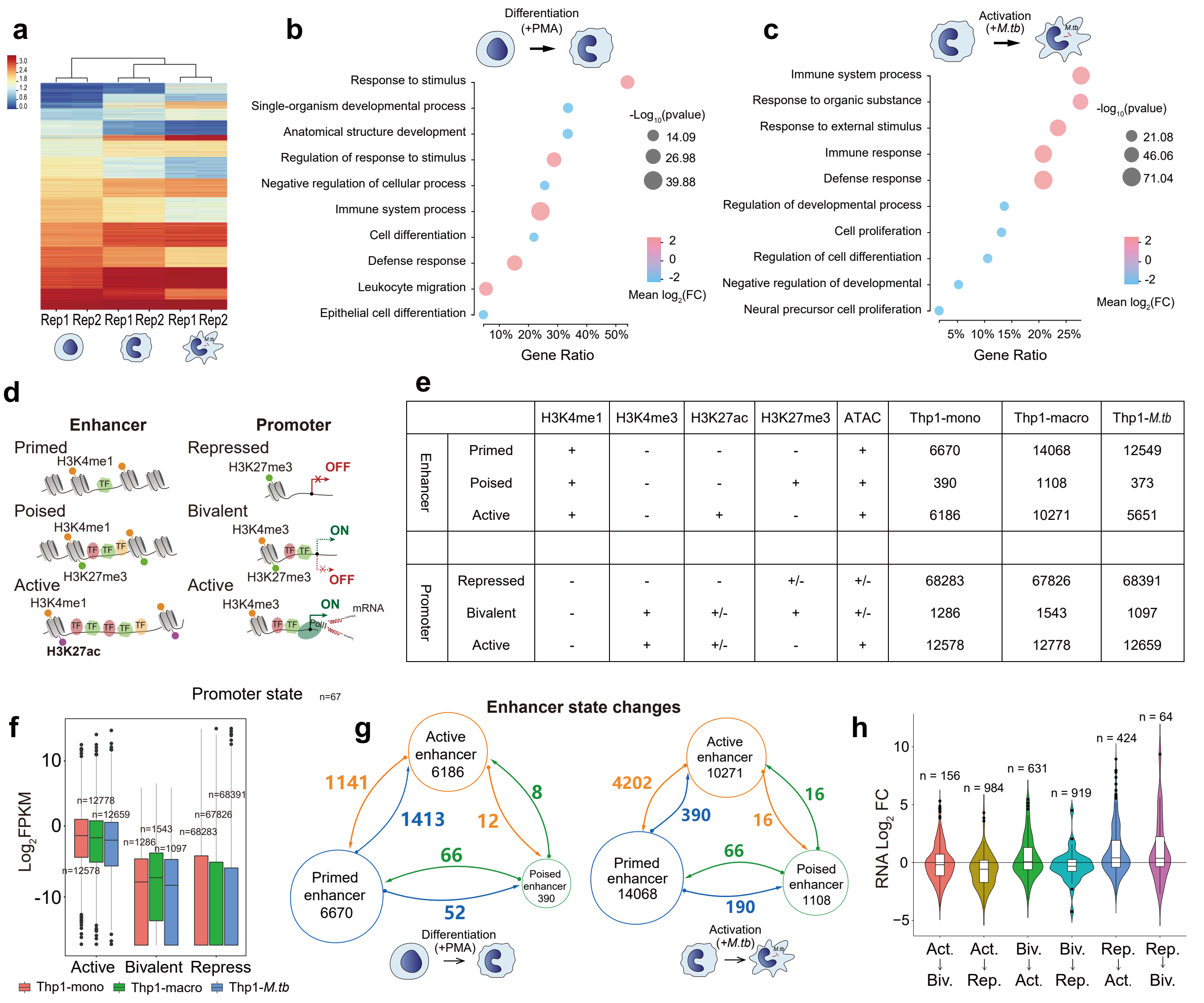

Supplementary Fig. 1 Chromatin state and transcription dynamics of THP-1 cells during differentiation and *M.tb* infection.

a) Clustering of gene expression profiles in THP-1 cells during differentiation and *M.tb* infection. The heatmap shows the relative expression levels of genes (rows) in each sample (column).

b,c) GO classification and statistical analysis of gene function during THP-1 cell differentiation and infection. P values were calculated by hypergeometric test.

d) Definition of enhancer and promoter states.

e) Different categories of enhancers and promoters identified among Thp1-mono, Thp1-macro, and Thp1-*M.tb* cells.

f) Comparison of gene expression levels between active, bivalent, and repressive promoters in different samples. Box-plot with midline = median, box limits = Q1 (25th percentile) / Q3 (75th percentile), whiskers = minimum and maximum values, points = outliers (> 1.5 inter-quartile range). The sample sizes (n) are labeled in the figure.

g) Dynamics of enhancer states during differentiation and *M.tb* infection. The arrows indicate the chromatin states changed from one state (tails of the arrows) to another state (heads of the arrows).

h) Effect of promoter status dynamics on gene expression during *M.tb* infection. “Act” indicates “Active promoter” , “Biv” indicates “Bivalent promoter” , “Rep” indicates “Repress promoter” . Box-plots inside each violin describe the interquartile range. Box-plot with midline = median, box limits = Q1 (25th percentile) / Q3 (75th percentile), whiskers = minimum and maximum values, points = outliers (> 1.5 inter-quartile range). The sample sizes (n) are labeled in the figure.

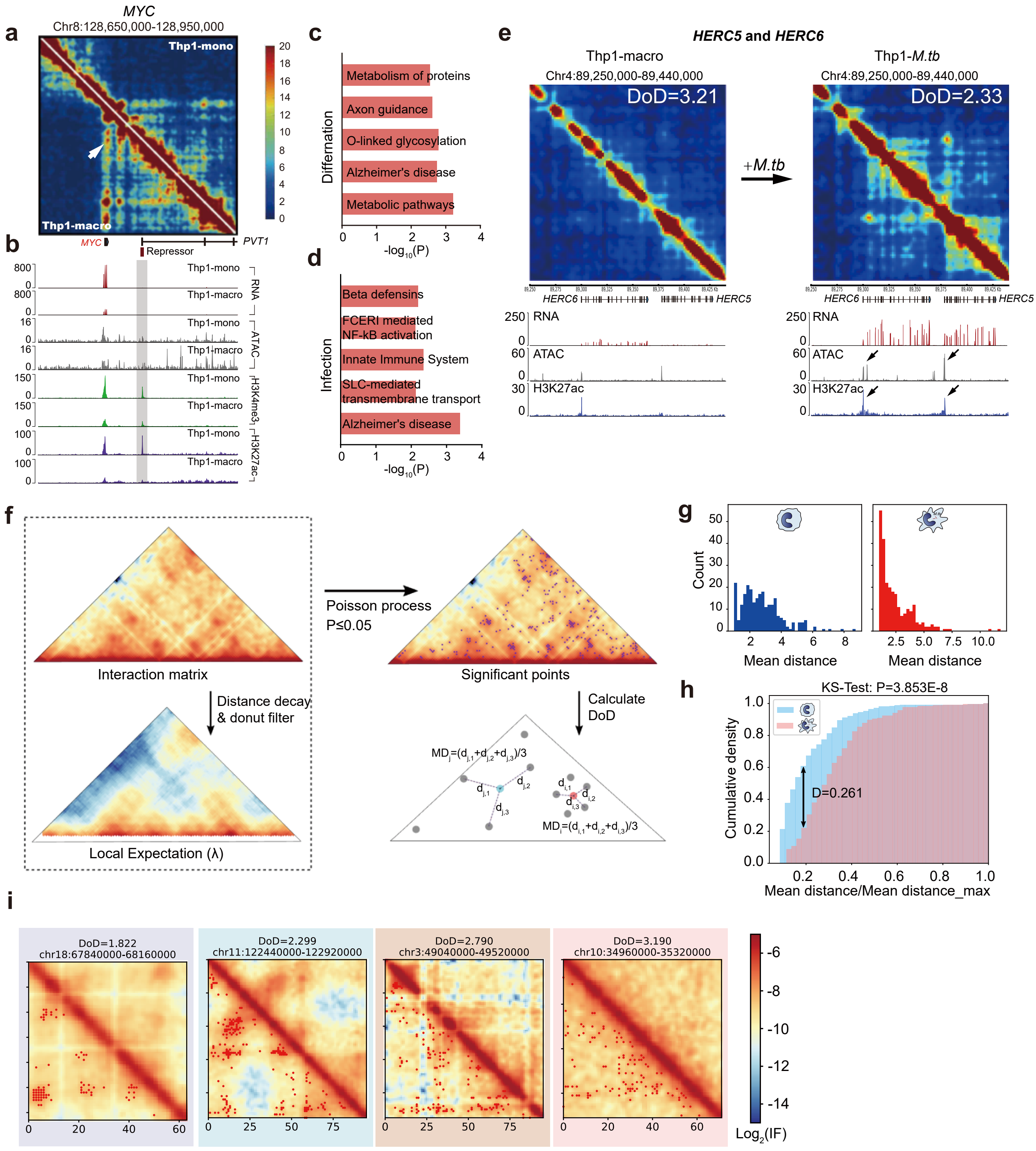

Supplementary Fig. 2 Calculation of TAD degree of disorder (DoD) in three types of cells.

a,b) Comparison of the chromosome interaction matrices, gene expression levels, and epigenetic modifications around *MYC* gene and repressor loci before and after THP-1 cell differentiation.

c,d) KEGG analysis of the genes around altered TAD boundaries during the differentiation (**c**) and activation (**d**) processes, KEGG analysis used the ClueGO plug-in in Cytoscape software (Version: 2.5.8). P values were calculated by hypergeometric test.

e) Compare chromatin contact matrix, RNA expression level, chromatin accessibility, H3K27ac histone modification changes around *HERC5* and *HERC6* gene region after *M.tb* infection. The significantly strengthened peaks were marked with arrow.

f) The illustration of computational pipeline for determining the TAD DoD. First, the local expectation of chromatin interactions was estimated using the distance decay function and donut filter. Then, the p-value of observing interaction in the contact matrix was calculated and screened based on the Poisson process. Interactions with P-value ≤ 0.05 are retained. The average distance to each significant point's k-nearest neighbors was designated as the MD (mean distance). The TAD DoD was calculated as the average value of the MD of all points.

g) Histogram showing the MD distribution before and after *M.tb* infection in the *GBP* region (Chr1:89,360,000-89,920,000).

h) The cumulative histogram used in the two-side KS (Kolmogorov-Smirnov) test for statistical comparison of the MD distribution in (**g**); here, the D statistic was equal to 0.261, and the p-value was equal to 3.852E-8.

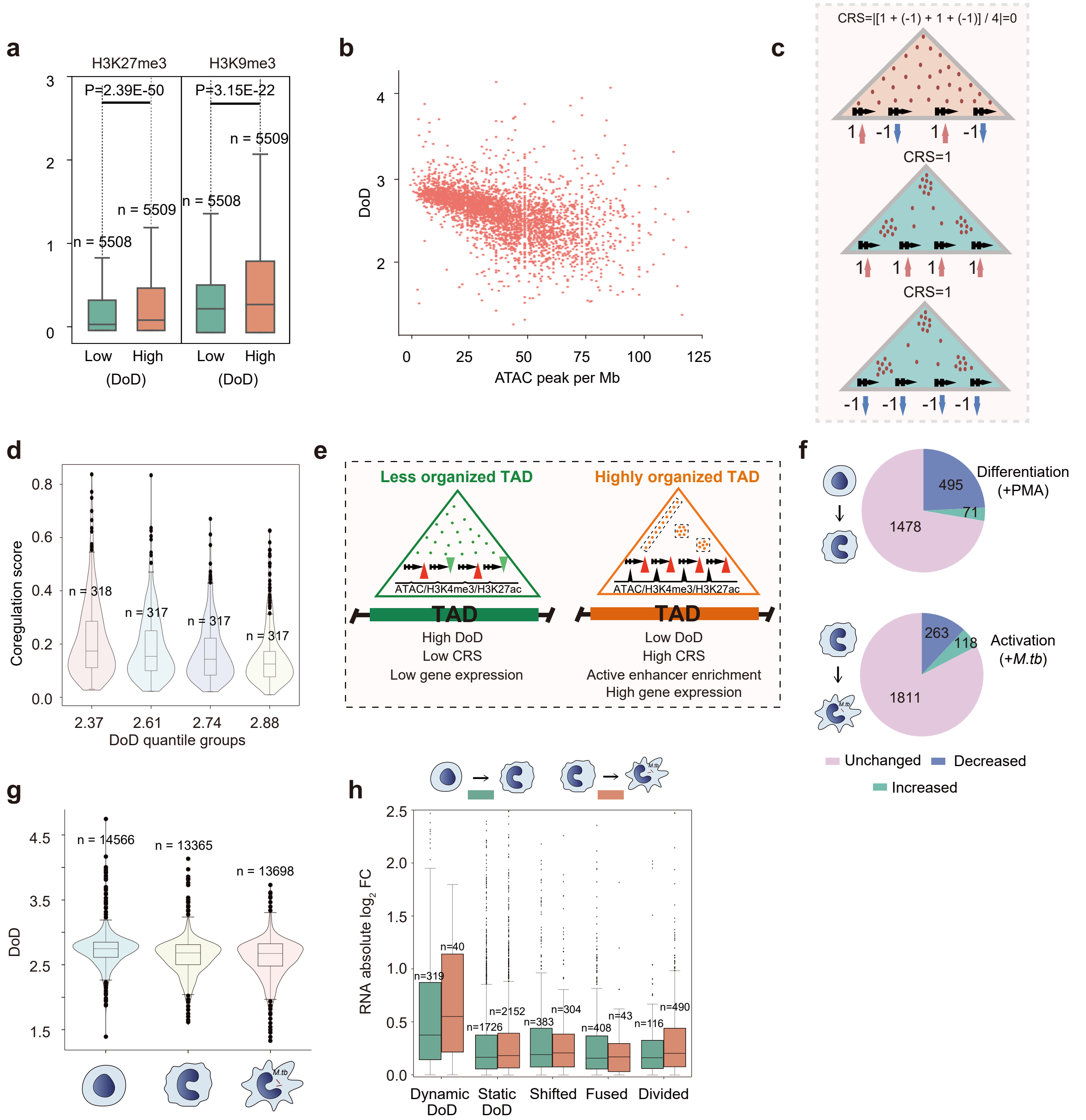

Supplementary Fig. 3 Correlation between TAD DoD and gene coregulation.

a) Correlation between TAD DoD and repress chromatin epigenetic profile. The y axis shows the normalized ChIP-seq peaks per TAD. The DoD value higher than median was defined as “High DoD” ; lower than the median was defined as “Low DoD” . Box-plot with midline = median, box limits = Q1 (25th percentile) / Q3 (75th percentile), whiskers = minimum and maximum values, points = outliers (> 1.5 inter-quartile range). The sample sizes (n) are labeled in the figure. Significant differences were measured by unpaired one-sided t test.

b) Correlation between TAD DoD and chromatin accessibility. Each dot represents one TAD. x-axis is the number of ATAC peaks per Mb.

c) Schematic illustrating the calculation of gene coregulation score (CRS) in TAD.

d) Correlation between the TAD DoD and coregulation score (CRS). x axis is the average value of the quantile groups with the lowest, low, high and highest TAD DoDs, respectively. Box-plots inside each violin describe the interquartile range. Box-plot with midline = median, box limits = Q1 (25th percentile) / Q3 (75th percentile), whiskers = minimum and maximum values, points = outliers (> 1.5 inter-quartile range). The sample sizes (n) are labeled in the figure.

e) The relationship between DoD value, chromatin folding order, chromatin accessibility, gene transcription coregulation, and histone modification.

f) DoD dynamics of boundary intact TADs during differentiation and infection.

g) Distribution of the DoD value of boundary unchanged TADs in Thp1-mono, Thp1-macro, and Thp1-*M.tb* cells. Box-plots inside each violin describe the inter-quartile range. Box-plot with midline = median, box limits = Q1 (25th percentile) / Q3 (75th percentile), whiskers = minimum and maximum values, points = outliers (> 1.5 inter-quartile range). The sample sizes (n) are labeled in the figure.

h) Absolute values of the log_2 fold changes in gene expression (y-axis) in the boundary not changed but DoD value significant changed TADs (dynamic DoD), boundary and DoD unchanged TADs (static DoD), boundary shifted TADs (shifted), boundary fused TADs (fused), and boundary divided TADs (divided) during differentiation and infection. Box-plot with midline = median, box limits = Q1 (25th percentile) / Q3 (75th percentile), whiskers = minimum and maximum values, points = outliers (> 1.5 inter-quartile range). The sample sizes (n) are labeled in the figure.

i) Examples of TADs with different TAD DoDs. From left to right, the TAD DoD value is increasing.

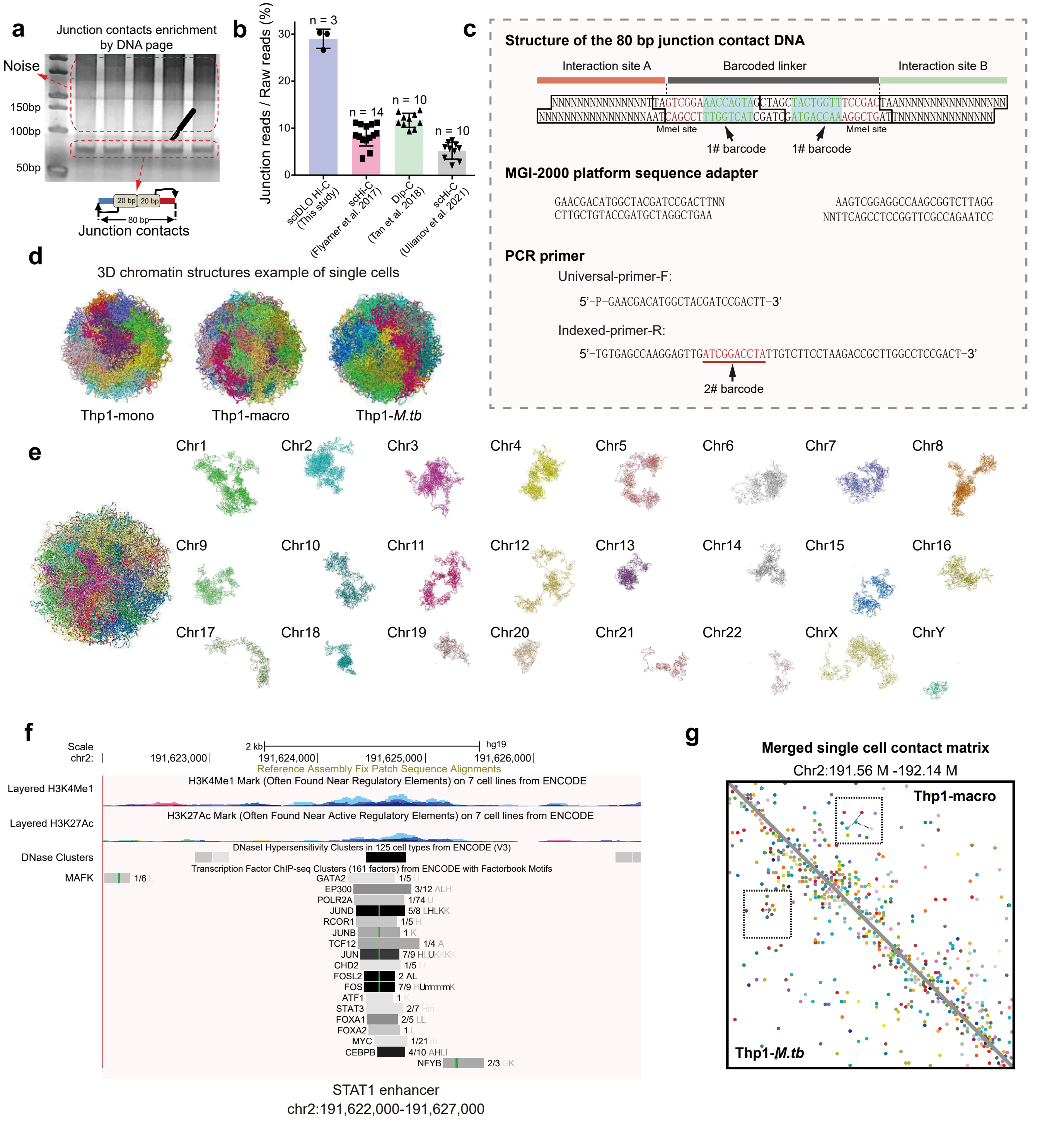

Supplementary Fig. 4 Structure of single cell indexed (sci) DLO Hi-C library.

a) Recovery of 80 bp proximity ligation junction DNA by DNA PAGE gel during sciDLO Hi-C. Each sample was replicated 3 times.

b) Junction reads between different single cell Hi-C data sets. Error bars show mean ± SD. The sample sizes (n) are labeled in the figure.

c) The structures of sequence adapter, 80 bp junction contact DNA and amplification primers used in sciDLO Hi-C experiment.

d) Simulated single cell 3D genome structure of Thp1-mono, Thp1-macro, and Thp1-*M.tb* cells at 25 kb resolution by using PyMOL software (2.3.0).

e) Identifying chromatin territories by using simulated single cell nucleus.

f) Histone modification, DNase clusters, and transcription factor enrichment in the *STAT1* enhancer region. The transcription factors regulating the *PD-L1* gene are marked by arrows. Data are from the UCSC Genome browser (http://genome.ucsc.edu).

g) Comparison of the single cell chromatin contact differences using merged single cell chromatin contact matrix. Each color of the dots in the matrix represents the chromatin contact from the same cell. The cells shown in Figure 3j-m are marked with dashed lines.

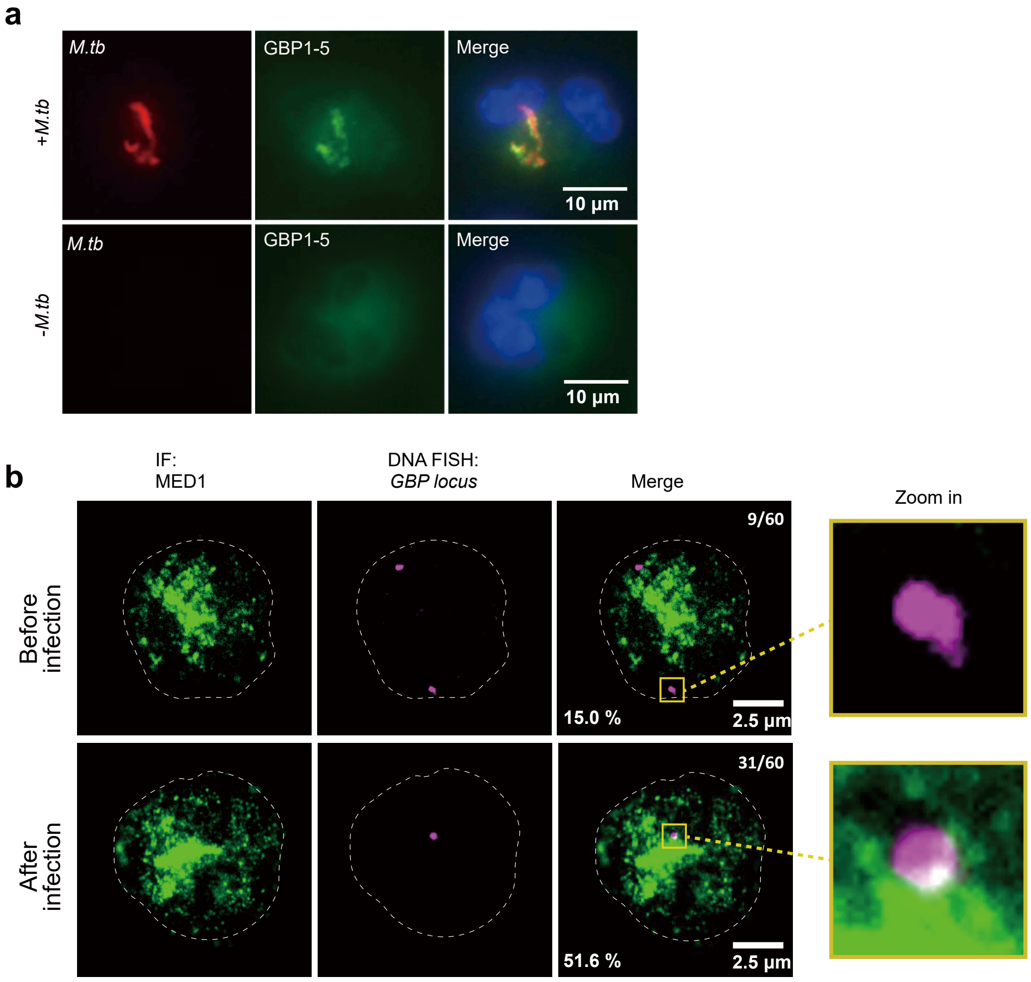

Supplementary Fig. 5 Verify the co-location of *GBP* gene cluster and MED1 protein.

a) Immunofluorescence (IF) analysis of GBP protein distribution around *M.tb* in THP-1 macrophages. Each experiment was replicated three times.

b) Co-localization between MED1 and the *GBP* gene cluster by IF and DNA-FISH in Thp1-macro and Thp1-*M.tb* cells. IF, DNA-FISH, and merged channels (overlapping signal in white) are shown in separate images. The dashed line highlights the nuclear periphery, determined by DAPI staining. The rightmost column shows the area in the yellow box in greater detail. For each cell type, we counted 60 *GBP* loci, 15.0 % (9/60) *GBP* loci were co-located with MED1 in Thp1-macro cells and 51.7 % (31/60) *GBP* loci were co-located with MED1 in Thp1-*M.tb* cells. Each experiment was replicated three times.

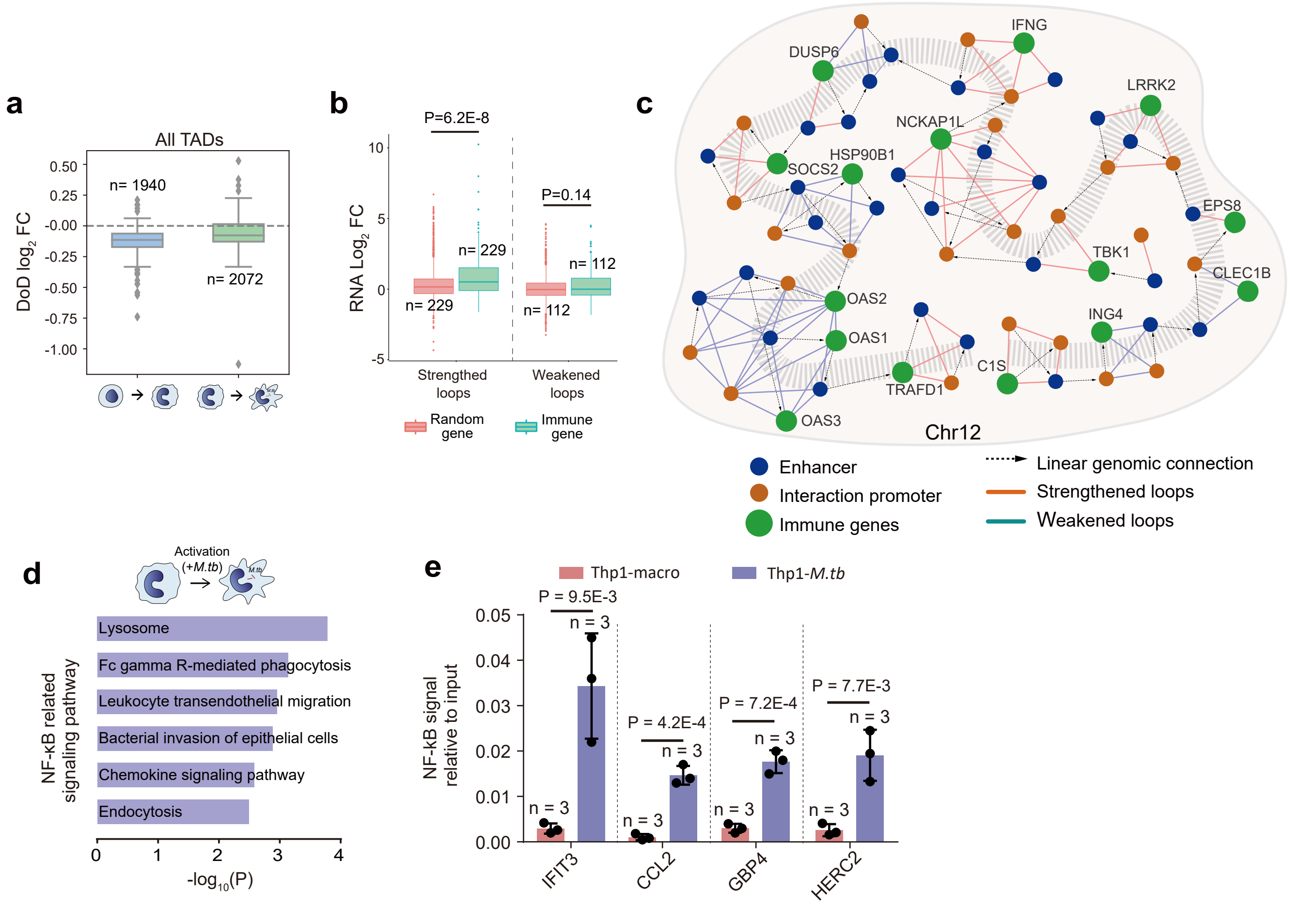

Supplementary Fig. 6 Transcription factor NF-κB is involved in the strengthening of target chromatin loops during *M.tb* infection.

a) Log_2 fold changes of overall TAD DoD value of THP-1 cells during differentiation and infection. Box-plot with midline = median, box limits = Q1 (25th percentile) / Q3 (75th percentile), whiskers = minimum and maximum values, points = outliers (> 1.5 inter-quartile range). The sample sizes (n) are labeled in the figure.

b) Log_2 RNA expression fold changes of the immune genes which located in the strengthened (immune gene, n= 229) and weakened (immune gene, n=112) loop anchors. Significant differences were calculated by unpaired one-sided t test. Box-plot with midline = median, box limits = Q1 (25th percentile) / Q3 (75th percentile), whiskers = minimum and maximum values, points = outliers (> 1.5 inter-quartile range). The sample sizes (n) are labeled in the figure.

c) Dynamic innate immune gene-regulatory element chromatin interaction network during *M.tb* infection.

d) KEGG pathway enrichment analysis of the genes located in strengthened loop anchors. P values were calculated by hypergeometric test.

e) ChIP-qPCR validation of the NF-κB (P65) enrichment on the loop anchor of the NF-κB target genes. The amount of immunoprecipitated DNA in each sample is represented as signal relative to the total amount of input chromatin (y-axis). Error bars show mean ± SD, n = 3 biologically independent repeats. P values were calculated by two-sided Student's t-test.

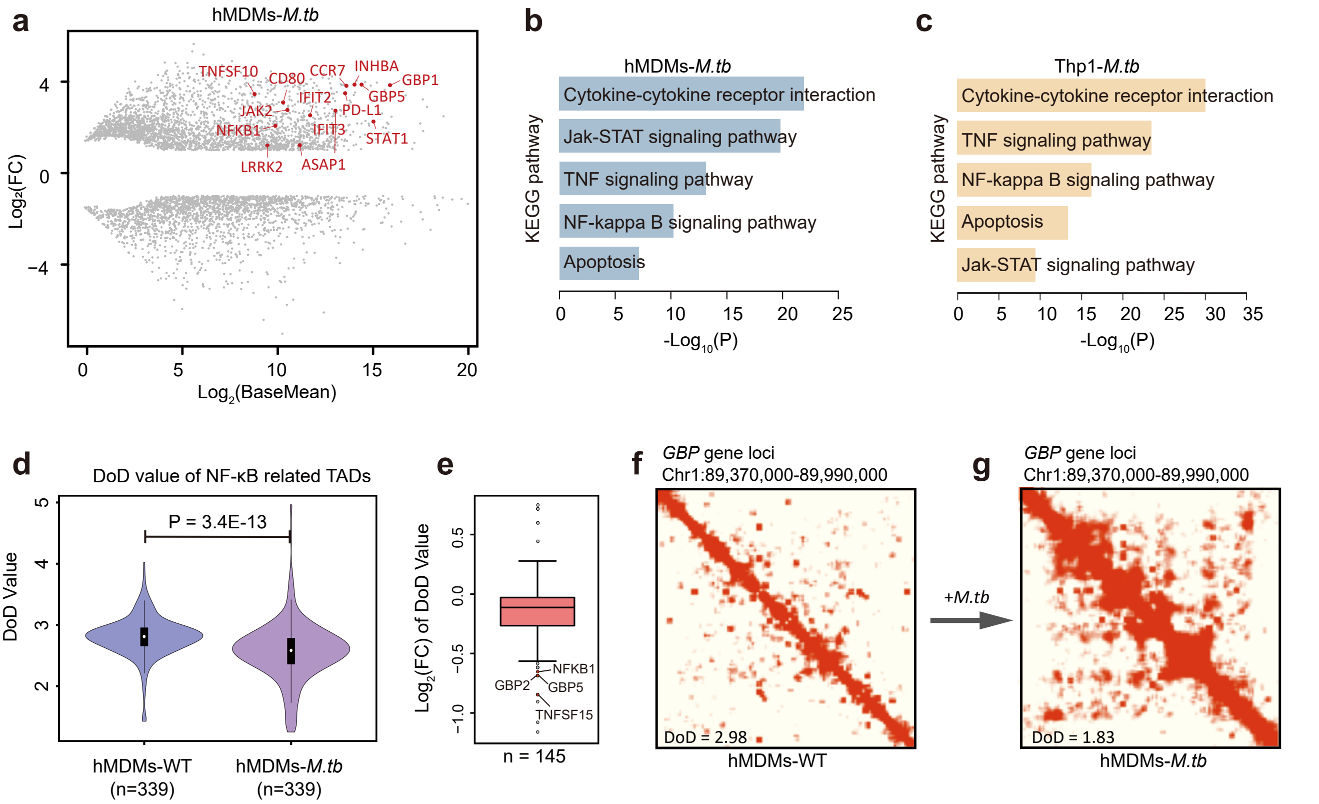

Supplementary Fig. 7 Gene expression and DoD changes of human monocyte derived macrophages (hMDMs) after *M.tb* infection.

a) MA plot for gene differential expression analysis during virulent tuberculosis strain H37Rv infection. X axis represents the mean of normalized counts, Y axis represents the log_2 fold changes of gene expression level. The immunity genes which were significantly upregulated in the Thp1-*M.tb* were marked in the figure.

b,c) KEGG pathway enrichment analysis for the differentially expressed genes. P values were calculated by hypergeometric test.

d) TAD DoD dynamics of NF-κB target loci after H37Rv infection in hMDM cells. P values were calculated by unpaired one-sided t test. Box-plots inside each violin describe the interquartile range. Box-plot with midline = median, box limits = Q1 (25th percentile) / Q3 (75th percentile), whiskers = minimum and maximum values. The sample sizes (n) are labeled in the figure.

e) Log_2 fold changes of TAD DoD value of NF-κB target locus after H37Rv infection in hMDM cells. The typical NF-κB target gene were marked in the figure. Box-plot with midline = median, box limits = Q1 (25th percentile)/Q3 (75th percentile), whiskers = minimum and maximum values, points = outliers (> 1.5 inter-quartile range). The sample sizes (n) are labeled in the figure.

f, g) Comparison of TAD DoD value changes around the *GBP* gene loci in hMDM cells before and after H37Rv infection.

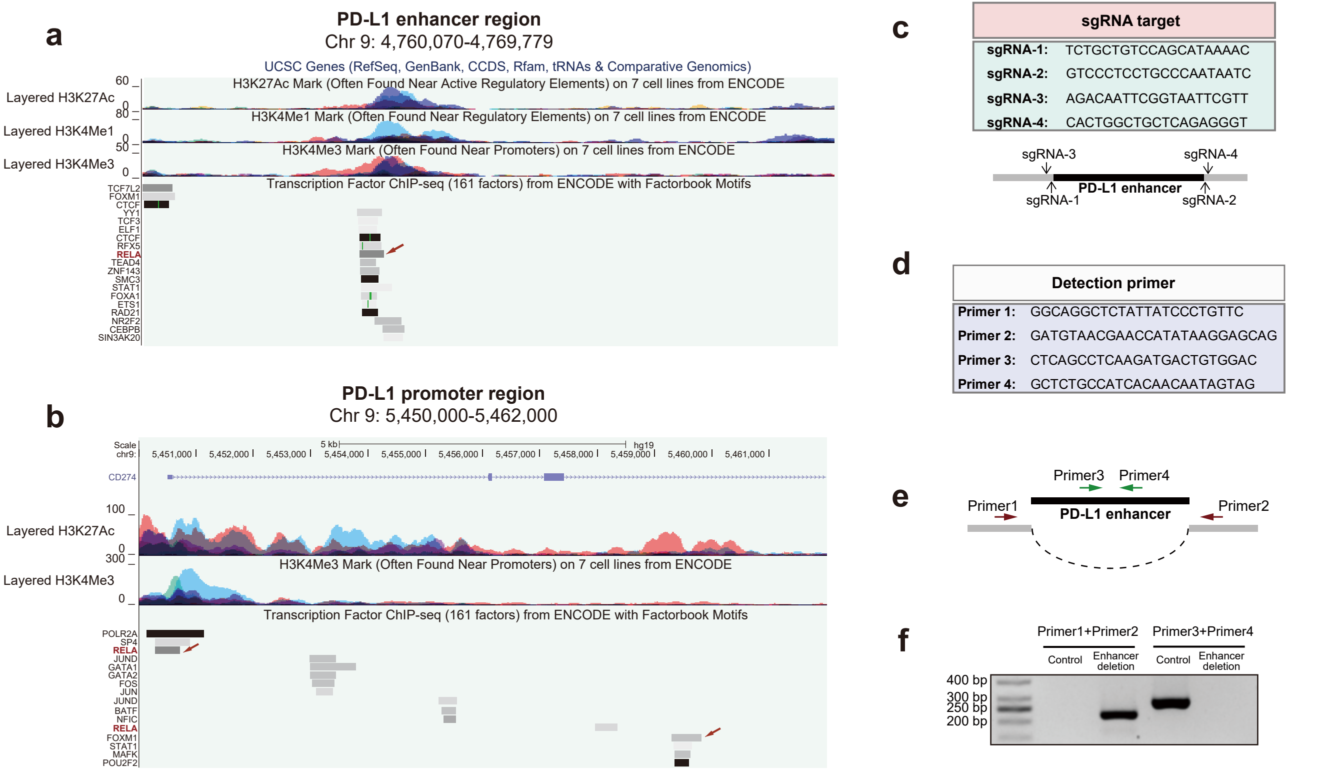

Supplementary Fig. 8 *PD-L1* enhancer identification and knockout.

a,b) Histone modification and transcription factor enrichment in the *PD-L1* promoter and enhancer region. The transcription factors regulating the *PD-L1* gene are marked by arrows. Data are from the UCSC Genome browser (http://genome.ucsc.edu).

c) sgRNAs used in *PD-L1* enhancer deletion. Two sgRNAs were designed for each side of the *PD-L1* enhancer.

d,e) Validation of homozygous *PD-L1* enhancer deletion.

f) Genotype of wild-type and *PD-L1* enhancer-deleted THP-1 cells as determined by PCR. Each experiment was repeated three times.

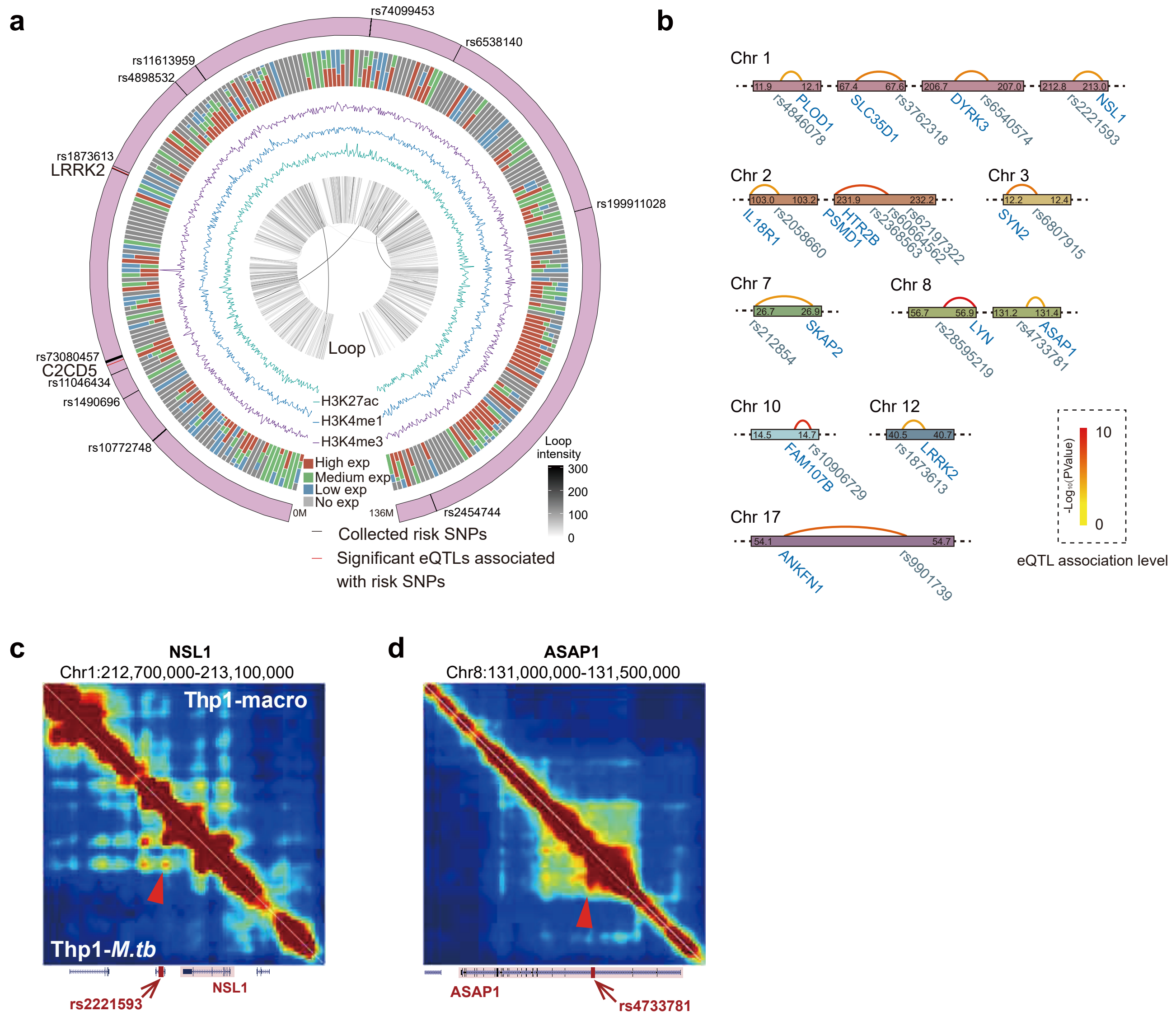

Supplementary Fig. 9 Comprehensive map of potential mycobacterial disease susceptibility genes by integrated eQTL, Hi-C and GWAS multi-omics analysis.

a) Circos plot of all reported mycobacterial disease risk SNPs and significant eQTLs associated with risk SNPs, with the corresponding gene expression levels, histone modifications and Hi-C loops on chromosome 12.

b) Identification of target genes of mycobacterial disease susceptibility SNPs by integrated eQTL, Hi-C and GWAS multi-omics analysis.

c,d) Comparison of the dynamic chromatin loops around the potential targets *NSL1* (**c**) and *ASAP1* (**d**) before and after *M.tb* infection. The strengthened loops and mycobacterial disease susceptibility SNPs are highlighted by arrow.

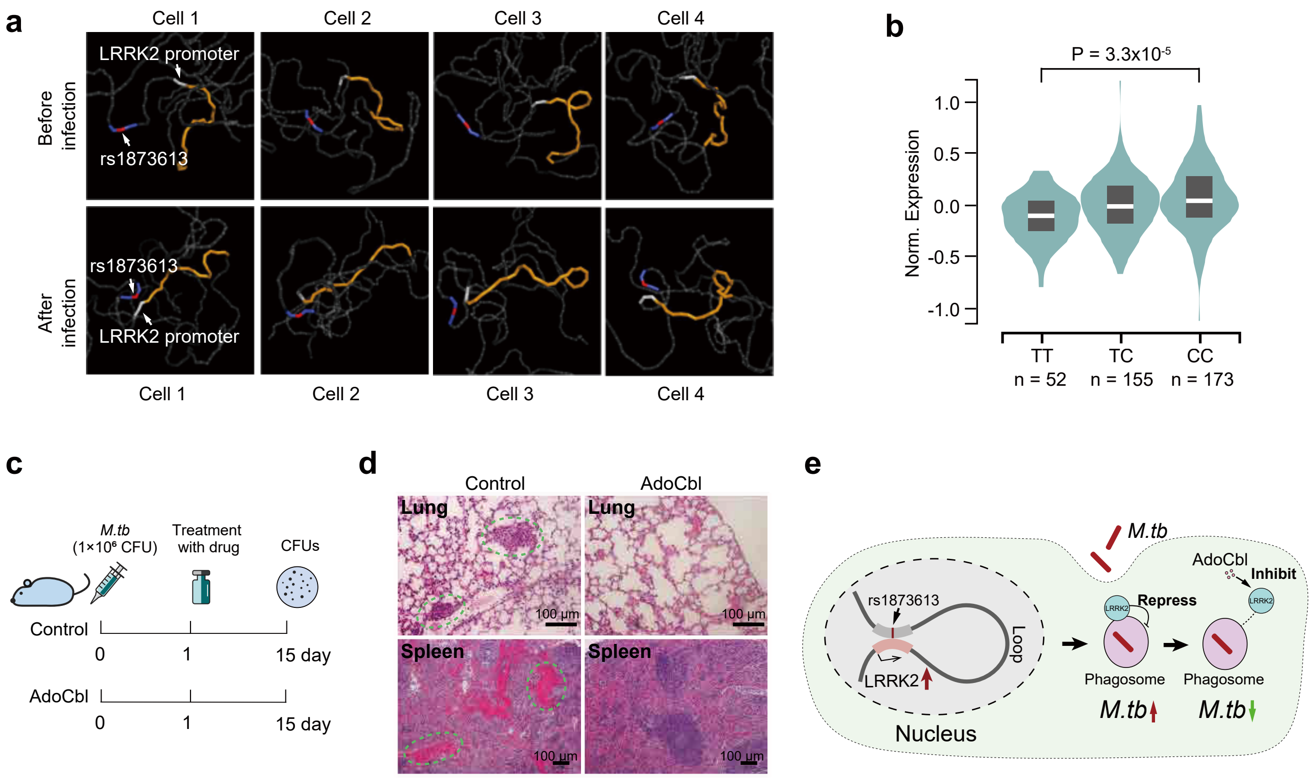

Supplementary Fig. 10 The possible mechanism by which the rs1873613 SNP causes TB susceptibility.

a) Simulated typical single cell chromatin structure of SNP rs1873613 and *LRRK2* gene region before and after *M.tb* infection by using PyMOL software (version: 2.3.0).

b) Regulation of *LRRK2* gene expression by the rs1873613. The x axis indicates three genotypes of individuals from the Genotype-Tissue Expression (GTEx) dataset. Box-plots inside each violin describe the interquartile range. Box-plot with midline = median, box limits = Q1 (25th percentile) / Q3 (75th percentile). P values were calculated by unpaired one-sided t test. The sample sizes (n) are labeled in the figure.

c) The time points of *M.tb* infection, drug treatment, and CFU analysis of animal experiment.

d) HE-stained lung and spleen sections in the AdoCbl and control (PBS) groups. Obvious lesions in the lungs and spleen are highlighted by the dashed lines. Each sample was replicated three times.

e) The possible mechanism by which the rs1873613 SNP causes TB susceptibility and the role of AdoCbl in the treatment of TB.

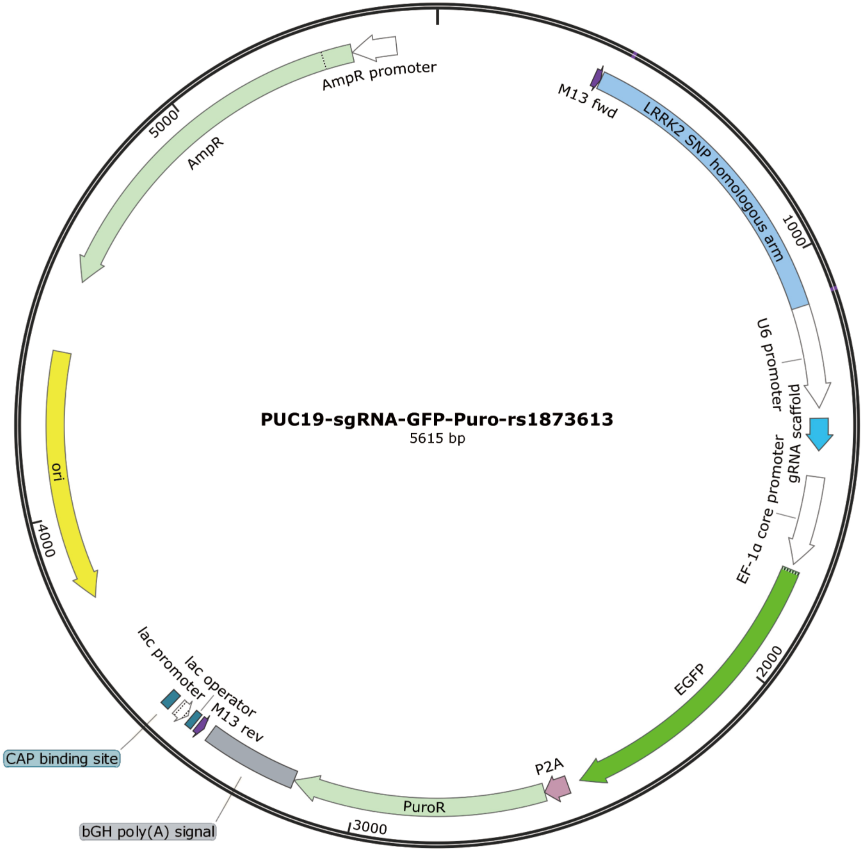

Supplementary Fig. 11 The structure of plasmid used to introduce rs1873613 mutation to THP-1 cells. This plasmid contains rs1873613 homologous DNA fragment, sgRNA cassette, GFP fluorescent protein gene, and puromycin-resistance gene.

	***In situ* DLO Hi-C library**											
**Category**	**Thp1-mono (rep1)**	**Thp1-mono (rep2)**	**Thp1-mono (rep3)**	**Thp1-mono (rep4)**	**Thp1-macro (rep1)**	**Thp1-macro (rep2)**	**Thp1-macro (rep3)**	**Thp1-macro (rep4)**	**Thp1-*M.tb* (rep1)**	**Thp1-*M.tb* (rep2)**	**Thp1-*M.tb* (rep3)**	**Thp1-*M.tb* (rep4)**
Raw reads	402,394,454	431,193,228	404,930,634	396,861,393	86,795,287	525,313,999	549,701,392	494,859,668	497,937,086	414,524,970	198,605,877	285,415,753
Linker reads	346,037,389	368,506,759	347,617,567	344,780,045	79,565,621	470,857,959	491,766,800	442,347,661	191,813,173	296,587,025	131,690,728	194,060,776
Uniquely-mapped reads	185,700,855	219,748,253	208,119,961	204,834,793	48,591,328	283,964,202	299,267,380	269,065,748	176,159,679	146,807,933	62,843,340	94,502,318
Non-redundant mapped reads	171,081,896	197,184,976	185,305,738	185,463,529	45,325,073	257,599,349	276,191,332	244,971,476	41,902,308	136,507,060	58,916,427	88,598,413
Inter chromosomal	57,481,075	65,678,111	61,014,521	62,416,540	11,452,083	65,629,389	70,640,711	63,131,789	134,257,371	33,063,110	13,716,838	20,109,793
Intra chromosomal	113,600,821	131,506,865	124,291,217	123,046,989	33,872,990	191,969,960	205,550,621	181,839,687	79,158,261	103,443,950	45,199,589	68,488,620
Intra long range (>5Kb)	102,290,003	115,952,498	109,424,226	108,830,819	27,344,158	154,908,928	166,158,775	147,124,914	103,043,263	79,480,234	34,714,276	52,695,396

Supplementary Table 1. Key performance metrics of different *in situ* DLO Hi-C library, including uniquely mapped reads (for evaluating mapping efficiency), non-redundantly mapped reads (for evaluating PCR redundancy), intra- or inter-chromosomal contact (for evaluating random ligation noise), and intra-short- or intra-long-range contact (for evaluating the noise of the dangling end and self-ligation reads).

Sample	Thp1-mono (96 x 14 barcodes)	Thp1-macro (96 x 14 barcodes)	Thp1-*M.tb* (96 x 14 barcodes)
Raw reads	363092708	372005843	330647901
Linker reads	224211951	220917116	202720351
Uniquely-mapped reads	110057271	111791351	88075112
Non-redundant mapped reads	26197299	29166452	28589317
Reads with two-round barcodes	21250507	21261922	24332690
Inter-chrom	1747724	1657716	2261886
Intra-chrom	19502783	19604206	22070804
Long-range contacts (>5 Kb)	3143816	2997536	3446247
Cells number (contacts> 10k)	409	424	510

Supplementary Table 2. Key performance metrics of merged sciDLO Hi-C library. In order to distinguish between single cells, we added two rounds of barcodes. In the first round, we added 96 barcodes to the 96-well plate, and in the second round, we added 14 barcodes in the sequencing adapter.
