# Supplementary material for: Decoding the spatial chromatin organization and dynamic epigenetic landscapes of macrophage cells during differentiation and immune activation
Source: Nat Commun. 2022 Oct 4;13:5857. doi: 10.1038/s41467-022-33558-5 (PMC9532393; doi:10.1038/s41467-022-33558-5)
Supplement: Supplementary file 3 — Description of additional Supplementary File [file 41467_2022_33558_MOESM3_ESM.pdf]

Descriptions of additional supplementary data files

Supplementary Data 1: Differentially expressed genes during THP-1 cell differentiation. The p-values were calculated by Wald test and adjusted (padj) by Benjamini and Hochberg method.

Supplementary Data 2: Differentially expressed genes during M.tb infection. The p-values were calculated by Wald test and adjusted (padj) by Benjamini and Hochberg method.

Supplementary Data 3 :Dynamics of promoter and enhancer states during THP-1 cell differentiation and M.tb infection.

Supplementary Data 4: Loop anchorlocated genes associated with chromatin remodeling during M.tb infection.

Supplementary Data 5: Integrated GWAS summary and eQTL in formation. The eQTL enrichment p-value was calculated by two-sided Fisher's exact test.

Supplementary Data 6: Barcoded linker, MGI-2000 platform sequence adapter, barcoded amplification primer, and sgRNA primer used in sciDLO Hi-C library construction.
