# Supplementary material for: Decoding the spatial chromatin organization and dynamic epigenetic landscapes of macrophage cells during differentiation and immune activation
Source: Nat Commun. 2022 Oct 4;13:5857. doi: 10.1038/s41467-022-33558-5 (PMC9532393; doi:10.1038/s41467-022-33558-5)
Supplement: Supplementary file 10 — Reporting Summary [file 41467_2022_33558_MOESM10_ESM.pdf]

Corresponding author(s): Gang Cao
Last updated by author(s): Sep 5, 2022

Reporting Summary

Nature Portfolio wishes to improve the reproducibility of the work that we publish. This form provides structure for consistency and transparency in reporting. For further information on Nature Portfolio policies, see our Editorial Policies and the Editorial Policy Checklist.

Statistics

For all statistical analyses, confirm that the following items are present in the figure legend, table legend, main text, or Methods section.

n/a	Confirmed	
☐	☒	The exact sample size (*n*) for each experimental group/condition, given as a discrete number and unit of measurement
☐	☒	A statement on whether measurements were taken from distinct samples or whether the same sample was measured repeatedly
☐	☒	The statistical test(s) used AND whether they are one- or two-sided<br>*Only common tests should be described solely by name; describe more complex techniques in the Methods section.*
☒	☐	A description of all covariates tested
☐	☒	A description of any assumptions or corrections, such as tests of normality and adjustment for multiple comparisons
☐	☒	A full description of the statistical parameters including central tendency (e.g. means) or other basic estimates (e.g. regression coefficient) AND variation (e.g. standard deviation) or associated estimates of uncertainty (e.g. confidence intervals)
☐	☒	For null hypothesis testing, the test statistic (e.g. *F*, *t*, *r*) with confidence intervals, effect sizes, degrees of freedom and *P* value noted<br>*Give P values as exact values whenever suitable.*
☒	☐	For Bayesian analysis, information on the choice of priors and Markov chain Monte Carlo settings
☒	☐	For hierarchical and complex designs, identification of the appropriate level for tests and full reporting of outcomes
☒	☐	Estimates of effect sizes (e.g. Cohen's *d*, Pearson's *r*), indicating how they were calculated

Our web collection on statistics for biologists contains articles on many of the points above.

Software and code

Policy information about availability of computer code

Data collection	No software was used for collection of genomic sequencing data.
Data analysis	FastQC (version: v0.11.8); Trimmomatic (version: 0.33); TopHat (version: v2.1.1); bowtie2 (version: 2.3.5.1); HTSeq (version: 0.11.2); DESeq2 (version: 1.36.0); SAMtools (version: 1.7); Burrows-Wheeler Aligner-MEM4 (version: 0.7.17-r1188); Picard toolkit (version: 1.119); F-Seq (version: 3); MACS2 (version: 2.1.1.20160309); scHiCTools (https://github.com/GeniusYx/scHiCTools, version: 0.0.3); DLO Hi-C tools (https://github.com/GangCaoLab/DLO-HiC-Tools, version: 0.3.9); Juicer Tools (version: 1.9.9); sciDLO Hi-C tools (https://github.com/GangCaoLab/sciDLO, version: 0.0.1); MDkNN (https://github.com/GangCaoLab/MDkNN, version: 0.0.1); nuc_dynamic (https://github.com/tjs23/nuc_dynamics, version: 1.3.0), PyMOL software (version: 2.3.0); DomainCaller (version: 0.1.0)

For manuscripts utilizing custom algorithms or software that are central to the research but not yet described in published literature, software must be made available to editors and reviewers. We strongly encourage code deposition in a community repository (e.g. GitHub). See the Nature Portfolio guidelines for submitting code & software for further information.

Data

Policy information about availability of data

All manuscripts must include a data availability statement. This statement should provide the following information, where applicable:

- Accession codes, unique identifiers, or web links for publicly available datasets
- A description of any restrictions on data availability
- For clinical datasets or third party data, please ensure that the statement adheres to our policy

All sequencing data generated in this study have been deposited in the Gene Expression Omnibus (GEO) under accession "GSE208046 [https://www.ncbi.nlm.nih.gov/geo/query/acc.cgi?acc=GSE208046]". The ChIP-seq data of NF-κB (GM15510 cell line) is download from ENCOCE Data Coordination Center (ENCODE DCC) [http://hgdownload.cse.ucsc.edu/goldenPath/hg19/encodeDCC/wgEncodeSydhTfbs].
The ChIP-seq data of MED1 and BRD4 is download from GEO under accession "GSE208046 [https://www.ncbi.nlm.nih.gov/geo/query/acc.cgi?acc=GSE160670]". The GWAS SNP was collected from the GWAS catalog [https://www.ebi.ac.uk/gwas] and UK Biobank GWAS datasets [http://www.nealelab.is/uk-biobank].
Source data for the figures and supplementary figures are provided as a Source Data file. Source data are provided with this paper.

Human research participants

Policy information about studies involving human research participants and Sex and Gender in Research.

Reporting on sex and gender	n/a
Population characteristics	n/a
Recruitment	n/a
Ethics oversight	n/a

Note that full information on the approval of the study protocol must also be provided in the manuscript.

Field-specific reporting

Please select the one below that is the best fit for your research. If you are not sure, read the appropriate sections before making your selection.

☒ Life sciences ☐ Behavioural & social sciences ☐ Ecological, evolutionary & environmental sciences

For a reference copy of the document with all sections, see nature.com/documents/nr-reporting-summary-flat.pdf

Life sciences study design

All studies must disclose on these points even when the disclosure is negative.

Sample size	Sample size were both indicated in the legend captions manuscript or the parametric results for all individuals for each sample plotted.
Data exclusions	No data were excluded from analyses
Replication	Experiments described in the manuscript were performed at least twice and all attempts at replication were successful.
Randomization	Mice were randomly divided into two equal groups before the M.tb infection experiment.
Blinding	The AdoCbl treatment group and control group was blinded to the investigator performing the CFU determinations.

Reporting for specific materials, systems and methods

We require information from authors about some types of materials, experimental systems and methods used in many studies. Here, indicate whether each material, system or method listed is relevant to your study. If you are not sure if a list item applies to your research, read the appropriate section before selecting a response.

Materials & experimental systems

n/a	Involved in the study
☐	☒ Antibodies
☐	☒ Eukaryotic cell lines
☒	☐ Palaeontology and archaeology
☐	☒ Animals and other organisms
☒	☐ Clinical data
☒	☐ Dual use research of concern

Methods

n/a	Involved in the study
☐	☒ ChIP-seq
☒	☐ Flow cytometry
☒	☐ MRI-based neuroimaging

Antibodies

Antibodies used

Antibody for ChIP-Seq

Name	Catalog number	Clone name	Company	Dilution
H3K4me1	ab8895	Polyclonal	Abcam	1:100
H3K4me3	ab8580	Polyclonal	Abcam	1:100
H3K9me3	ab8898	Polyclonal	Abcam	1:100
H3K27me3	07-449	Polyclonal	Millipore	1:100
H3K27ac	ab4729	Polyclonal	Abcam	1:100
NF-κB p65	8242	D14E12	CST	1:100

Antibody for IF

Name	Catalog number	Clone name	Company	Dilution
Rab7	sc-376362-AF488	B3	Santa Cruz	1:250
BRD4	ab128874	EPR5150(2)	Abcam	1:250
MED1	ab64965	Polyclonal	Abcam	1:250
GBP1-5	sc-166960 AF488	G-12	Santa Cruz	1:250
NF-κB p65	sc-8008 AF546	F6	Santa Cruz	1:250

Antibody for western

Name	Catalog number	Clone name	Company	Dilution
PD-L1	13684T	E1L3N	CST	1:1000
ACTIN	3700	8H10D10	CST	1:1000

Secondary antibodies

Name	Catalog number	Clone name	Company	Dilution
anti-rabbit HRP-conjugated	ab97080	polyclonal	Abcam	1:5000
anti-mouse HRP-conjugated	ab97046	polyclonal	Abcam	1:5000
Peroxidase AffiniPure Goat Anti-Mouse IgG (H+L)	A11008	polyclonal	Invitrogen	1:1000

Validation

Antibodies were purchased from reputable manufacturers, and selected in part based on usage in peer-reviewed publications:
H3K4me1,H3K4me3,H3K9me3,H3K27ac, and H3K27me3:
Zviran, Asaf, et al. "Deterministic somatic cell reprogramming involves continuous transcriptional changes governed by Myc and epigenetic-driven modules." Cell Stem Cell 24.2 (2019): 328-341. (DOI: 10.1016/j.stem.2018.11.014)
GBP1-5:
Sasai, Miwa, et al. "Essential role for GABARAP autophagy proteins in interferon-inducible GTPase-mediated host defense." Nature immunology 18.8 (2017): 899. (DOI: 10.1038/ni.3767)
Rab7:
Fedeli, Chiara, et al. "PSEN2 (presenilin 2) mutants linked to familial Alzheimer disease impair autophagy by altering Ca2+ homeostasis." Autophagy 15.12 (2019): 2044-2062. (DOI: 10.1080/15548627.2019.1596489)
NF-κB p65 :
Yamanaka, Satoshi, et al. "An IMiD-induced SALL4 degron system for selective degradation of target proteins." Communications biology 3.1 (2020): 1-14. (DOI: 10.1038/s42003-020-01240-5)
anti-BRD4 and anti-MED1:
Sabari, B. R. , Alessandra, D. , Ann, B. , Klein, I. A. , Coffey, E. L. , & Krishna, S. , et al. (2018). Coactivator condensation at super-enhancers links phase separation and gene control. Science, 361(6400), eaar3958-. (DOI: 10.1126/science.aar3958)

Eukaryotic cell lines

Policy information about cell lines and Sex and Gender in Research

Cell line source(s)

THP-1 (ATCC, TIB-202) cell lines were purchased from ATCC.

Authentication

The cell line was authenticated by whole-genome sequencing.

Mycoplasma contamination	The cell lines tested negative for mycoplasma contamination.
Commonly misidentified lines (See ICLAC register)	No commonly misidentified cell lines were used.

Animals and other research organisms

Policy information about studies involving animals; ARRIVE guidelines recommended for reporting animal research, and Sex and Gender in Research

Laboratory animals	All wild type female C57BL/6 mice used in this study were purchased from Beijing Vital River Laboratory Animal Technology and all the experiments in this study were approved by the Scientific Ethic Committee of Huazhong Agricultural University (NO. HZAUMO-2019-019) and maintained at the Laboratory Animal Centre of Huazhong Agriculture University under specific pathogen-free (SPF) conditions with 12-hour light/dark cycles. Room temperature was maintained at 25°C. The humidity level was controlled between 40-60%. Based on the principles of laboratory animal welfare and ethics, this study optimized the design of the project and strictly plans the number of animals required. A total of 28 6-week-old female C57BL/6 mice (20±2g) were planned. Among them, 4 were used for bone marrow macrophage isolation experiments, and the remaining 24 were used for Mycobacterium tuberculosis H37Ra infection test and AdoCbl drug treatment experiment.
Wild animals	No wild animals were used.
Reporting on sex	28 purchased C57BL/6 female mice were normal feeding for 7 days, and 4 euthanized mice were used to isolate bone marrow macrophages for in vitro Mycobacterium tuberculosis infection and AdoCbl drug experiments; the remaining 24 mice were infected by intravenous injection with a dose of 5×106 cfu/mL of 200μL M.tb H37Ra suspension. After infection, all animals were randomly distributed into 2 groups of 12 each. Then mice were received a gavage feeding of AdoCbl (Sigma, Cat# C0884) at a daily dose of 0.5 mg/kg·bw and equal volume of sterile PBS used as a negative control. These mice were given the therapy for 1 day post of M.tb infection. Mice were euthanized after 15 days of drug treatment. Lungs and spleen were taken out for histopathological observation and CFU analysis and make section for haematoxylin and eosin (H&E) staining.
Field-collected samples	No field collected samples were used in the study
Ethics oversight	HZAUMO-2019-019 approved by The Scientific Ethic Committee of Huazhong Agricultural University

Note that full information on the approval of the study protocol must also be provided in the manuscript.

ChIP-seq

Data deposition

☒ Confirm that both raw and final processed data have been deposited in a public database such as GEO.

☒ Confirm that you have deposited or provided access to graph files (e.g. BED files) for the called peaks.

Data access links *May remain private before publication.*	https://www.ncbi.nlm.nih.gov/geo/query/acc.cgi?acc=GSE208046
Files in database submission	1-H3K27me3-THP-07-449-1_R1.fastq.gz<br>1-H3K27me3-THP-07-449-1_R2.fastq.gz<br>10-H3K9me3-THP-8898-2_R1.fastq.gz<br>10-H3K9me3-THP-8898-2_R2.fastq.gz<br>11-input-THP_R1.fastq.gz<br>11-input-THP_R2.fastq.gz<br>2-H3K27me3-THP-07-449-2_R1.fastq.gz<br>2-H3K27me3-THP-07-449-2_R2.fastq.gz<br>3-H3K27ac-THP-4729-1_R1.fastq.gz<br>3-H3K27ac-THP-4729-1_R2.fastq.gz<br>4-H3K27ac-THP-4729-2_R1.fastq.gz<br>4-H3K27ac-THP-4729-2_R2.fastq.gz<br>5-H3K4me1-THP-8895-1_R1.fastq.gz<br>5-H3K4me1-THP-8895-1_R2.fastq.gz<br>6-H3K4me1-THP-8895-2_R1.fastq.gz<br>6-H3K4me1-THP-8895-2_R2.fastq.gz<br>7-H3K4me3-THP-8580-1_R1.fastq.gz<br>7-H3K4me3-THP-8580-1_R2.fastq.gz<br>8-H3K4me3-THP-8580-2_R1.fastq.gz<br>8-H3K4me3-THP-8580-2_R2.fastq.gz<br>9-H3K9me3-THP-8898-1_R1.fastq.gz<br>9-H3K9me3-THP-8898-1_R2.fastq.gz<br>1-H3K27me3-THPP-07-449-1_R1.fastq.gz<br>1-H3K27me3-THPP-07-449-1_R2.fastq.gz<br>10-H3K9me3-THPP-8898-2_R1.fastq.gz

10-H3K9me3-THPP-8898-2_R2.fastq.gz
11-input-THPP_R1.fastq.gz
11-input-THPP_R2.fastq.gz
2-H3K27me3-THPP-07-449-2_R1.fastq.gz
2-H3K27me3-THPP-07-449-2_R2.fastq.gz
3-H3K27ac-THPP-4729-1_R1.fastq.gz
3-H3K27ac-THPP-4729-1_R2.fastq.gz
4-H3K27ac-THPP-4729-2_R1.fastq.gz
4-H3K27ac-THPP-4729-2_R2.fastq.gz
5-H3K4me1-THPP-8895-1_R1.fastq.gz
5-H3K4me1-THPP-8895-1_R2.fastq.gz
6-H3K4me1-THPP-8895-2_R1.fastq.gz
6-H3K4me1-THPP-8895-2_R2.fastq.gz
7-H3K4me3-THPP-8580-1_R1.fastq.gz
7-H3K4me3-THPP-8580-1_R2.fastq.gz
8-H3K4me3-THPP-8580-2_R1.fastq.gz
8-H3K4me3-THPP-8580-2_R2.fastq.gz
9-H3K9me3-THPP-8898-1_R1.fastq.gz
9-H3K9me3-THPP-8898-1_R2.fastq.gz
1-H3K27me3-Ra-07-449-1_R1.fastq.gz
1-H3K27me3-Ra-07-449-1_R2.fastq.gz
10-H3K9me3-Ra-8898-2_R1.fastq.gz
10-H3K9me3-Ra-8898-2_R2.fastq.gz
11-input-Ra_R1.fastq.gz
11-input-Ra_R2.fastq.gz
2-H3K27me3-Ra-07-449-2_R1.fastq.gz
2-H3K27me3-Ra-07-449-2_R2.fastq.gz
3-H3K27ac-Ra-4729-1_R1.fastq.gz
3-H3K27ac-Ra-4729-1_R2.fastq.gz
4-H3K27ac-Ra-4729-2_R1.fastq.gz
4-H3K27ac-Ra-4729-2_R2.fastq.gz
5-H3K4me1-Ra-8895-1_R1.fastq.gz
5-H3K4me1-Ra-8895-1_R2.fastq.gz
6-H3K4me1-Ra-8895-2_R1.fastq.gz
6-H3K4me1-Ra-8895-2_R2.fastq.gz
7-H3K4me3-Ra-8580-1_R1.fastq.gz
7-H3K4me3-Ra-8580-1_R2.fastq.gz
8-H3K4me3-Ra-8580-2_R1.fastq.gz
8-H3K4me3-Ra-8580-2_R2.fastq.gz
9-H3K9me3-Ra-8898-1_R1.fastq.gz
9-H3K9me3-Ra-8898-1_R2.fastq.gz
1-H3K27me3-Ra-07-449-1.bigwig
1-H3K27me3-THP-07-449-1.bigwig
1-H3K27me3-THPP-07-449-1.bigwig
10-H3K9me3-Ra-8898-2.bigwig
10-H3K9me3-THP-8898-2.bigwig
10-H3K9me3-THPP-8898-2.bigwig
11-input-Ra.bigwig
11-input-THP.bigwig
11-input-THPP.bigwig
2-H3K27me3-Ra-07-449-2.bigwig
2-H3K27me3-THP-07-449-2.bigwig
2-H3K27me3-THPP-07-449-2.bigwig
3-H3K27ac-Ra-4729-1.bigwig
3-H3K27ac-THP-4729-1.bigwig
3-H3K27ac-THPP-4729-1.bigwig
4-H3K27ac-Ra-4729-2.bigwig
4-H3K27ac-THP-4729-2.bigwig
4-H3K27ac-THPP-4729-2.bigwig
5-H3K4me1-Ra-8895-1.bigwig
5-H3K4me1-THP-8895-1.bigwig
5-H3K4me1-THPP-8895-1.bigwig
6-H3K4me1-Ra-8895-2.bigwig
6-H3K4me1-THP-8895-2.bigwig
6-H3K4me1-THPP-8895-2.bigwig
7-H3K4me3-Ra-8580-1.bigwig
7-H3K4me3-THP-8580-1.bigwig
7-H3K4me3-THPP-8580-1.bigwig

8-H3K4me3-Ra-8580-2.bigwig
8-H3K4me3-THP-8580-2.bigwig
8-H3K4me3-THPP-8580-2.bigwig
9-H3K9me3-Ra-8898-1.bigwig
9-H3K9me3-THP-8898-1.bigwig
9-H3K9me3-THPP-8898-1.bigwig

Genome browser session
(e.g. UCSC)

UCSC

Methodology

Replicates

For each biological sample, we used 5 type of antibodies to carry out ChIP-seq experiment, and each antibody has two replicates.

Sequencing depth

1.ChIP-seq data of THP-1(Monocyte)

Name	length	Raw reads	Uniq map
H3K27ac_1	PE150	29,745,116	24,997,033
H3K27ac_2	PE150	33,441,319	28,109,059
H3K4me3_1	PE150	30,117,164	24,955,774
H3K4me3_2	PE150	33,108,437	27,890,678
H3K4me1_1	PE150	32,771,956	28,257,407
H3K4me1_2	PE150	21,944,383	18,920,250
H3K27me3_1	PE150	2,785,703	28,267,971
H3K27me3_2	PE150	33,603,112	28,543,669
H3K9me3_1	PE150	36,814,560	29,577,454
H3K9me3_2	PE150	33,368,457	26,913,232

2.ChIP-seq data of THP-1(Macrophage)

Name	length	Raw reads	Uniq map
H3K27ac_1	PE150	42,386,656	37,758,402
H3K27ac_2	PE150	48,712,742	43,422,814
H3K4me3_1	PE150	41,255,954	35,807,152
H3K4me3_2	PE150	44,922,240	39,133,456
H3K4me1_1	PE150	38,719,270	34,837,162
H3K4me1_2	PE150	30,001,812	27,028,139
H3K27me3_1	PE150	24,957,760	22,285,667
H3K27me3_2	PE150	36,778,179	32,684,974
H3K9me3_1	PE150	44,074,510	37,062,426
H3K9me3_2	PE150	38,942,897	32,642,486

3.ChIP-seq data of THP-1(M.tb infected macrophage)

Name	length	Raw reads	Uniq map
H3K27ac_1	PE150	19,958,585	16,344,972
H3K27ac_2	PE150	18,494,175	14,803,569
H3K4me3_1	PE150	22,942,353	18,194,885
H3K4me3_2	PE150	19,136,097	15,395,286
H3K4me1_1	PE150	20,814,133	16,944,904
H3K4me1_2	PE150	18,704,255	15,156,256
H3K27me3_1	PE150	19,728,818	16,156,734
H3K27me3_2	PE150	18,271,107	15,011,988
H3K9me3_1	PE150	18,876,050	14,664,631
H3K9me3_2	PE150	18,189,946	14,166,512

Antibodies

H3K4me1 (Abcam, ab8895); H3K4me3(Abcam, ab8580);H3K9me3 (Abcam, ab8898); H3K27ac (Abcam, ab4729); H3K27me3 (Millipore, 07-449).

Peak calling parameters

The reads with the mapping quality higher than 30 were considered as uniquely mapped reads. Peaks were called by MACS246 (version: 2.1.1.20160309). For broad peaks, the parameters were -B --broad -q 0.05. For narrow peaks, the parameter was -B.

Data quality

Fastqc was used to assess data quality. All the ChIP-seq peaks used for analysis were >20-fold enriched over input control libraries.

Software

Burrows-Wheeler Aligner-MEM (version: 0.7.17-r1188); SAMtools (version: 701 1.7); MACS2 (version: 2.1.1.20160309).
